# South Africa’s capacity to conduct antimicrobial stewardship

**DOI:** 10.4102/sajid.v36i1.297

**Published:** 2021-09-28

**Authors:** Sarentha Chetty

**Affiliations:** 1 Department of Pharmacy and Pharmacology, Faculty of Health Sciences, University of the Witwatersrand, Johannesburg, South Africa; 2 Department of Pharmacy, Faculty of Health Sciences, University of KwaZulu-Natal, Durban, South Africa

Antimicrobial resistance (AMR) as a serious public health threat was globally acknowledged by WHO in 2015, through the launch of the Global Action Plan (GAP).^1^ With a limited number of new antibiotics in the developmental pipeline, many countries are in the process of establishing strategies for antimicrobial stewardship (AMS).^1^ Within each country, different healthcare challenges have contributed to AMR. This has also shaped individual AMS strategies and policies. In South Africa (SA), there is a high burden of infectious diseases, mainly of bacterial origin.^2^ In addition, SA also has the highest number of people living with human immunodeficiency virus (HIV) globally.^2^ According to the 2019 statistics, there are approximately 7.97 million people living with HIV in SA. Together with this, SA has the fourth largest tuberculosis population globally.^2^ Other important challenges include poverty, malnutrition, a high burden of non-communicable diseases, and a dire shortage of trained healthcare professionals (e.g. clinicians, pharmacists, and nurses).^3^

Antimicrobial use in SA is 21 149 standard units per 1000/population. Standard units per 1000/population is the amount of antimicrobials supplied by pharmaceutical manufacturers to the public and private sector. This is comparable to use in other BRICS (Brazil, Russia, India, China, and South Africa) countries but is higher than non-BRICS countries. Broad spectrum antibiotics are 1.3–3.3 times more likely to be used in SA than in other BRICS countries and South Africa has a 0.8 times higher broad spectrum antimicrobial usage than the United Kingdom (UK) or the United States of America (USA).^4^ In 2017, ESKAPE organisms accounted for 33% (*n* = 22 788) of all positive cultures of blood samples sent to the National Health Laboratory Service (NHLS) from the public sector.^4^ A recent study in 26 healthcare facilities throughout SA showed that the average compliance to the SA AMR national strategic framework was 59.5%. The results revealed the following compliance levels: community health centres (38%), referral hospitals (66.9%), and national central hospitals (73.5%). Only five facilities had a compliance greater than 80% and seven were recorded to have a compliance of less than 50%.^5^

The newest public health threat, that is the emergence of coronavirus disease 2019 (COVID-19) pandemic threatens to derail the efforts to curb antimicrobial use. With a steady rise in the daily COVID-19 infections around the world, there have been reports of widespread global antimicrobial use. Although antimicrobial prescribing practices differ from country to country,^6,7^ it is now well recognised that this global pandemic will further accelerate AMR.^6,7,8^ Now more than ever, the importance of establishing antimicrobial stewardship programmes (ASP) in the country cannot be overemphasised.

A paucity of data exists to ASP implementation on the African continent. Published literature, however, suggests that SA in comparison to other African countries has made commendable strides in AMS implementation.^9^ A recent scoping review focussing on AMS in South Africa revealed that many hospitals in both the public and private sector across the country were involved in some form of AMS activity, but most of these were basic quality improvement projects. In the private sector, AMS activities were often implemented across hospital groups. In the public sector, however, AMS implementations were only successfully accomplished in a few hospitals.^10,11,12,13^

The results of a recent situational analysis of AMS activities in the KwaZulu-Natal (KZN) public sector hospital facilities paints a similar picture (‘unpublished data’). Although a vast majority of hospitals had set up governance structures, including AMS committees and a term of reference, individual stewardship programmes were at different levels of functioning. The main challenges cited were lack of finance, IT, microbiology and infectious diseases specialists support. Time and human resource shortages was another aspect that affected the institution’s ability to successfully run a stewardship programme (‘unpublished data’).

This finding begs the question of whether the current resources, capacities and leadership support are sufficient to run these programmes at a higher scale or capacity.

In resource rich countries, AMS teams are typically multidisciplinary consisting of clinical pharmacists, infectious disease specialists and clinical microbiologists. All of whom have been adequately trained in the principles of stewardship.^14,15^ In SA, as in low and middle income countries (LMICs), this is not always the case. Often facilities have had to leverage the restricted available resources to provide a limited service making it only possible to accomplish the low-hanging fruit of AMS.^14,16^ Few examples of fully fledged ASPs are present in the public sector and success of these ASPs can be directly attributable to a multidisciplinary team and dedicated time and resources.^10,11,12,13^ In the private sector, ASPs are more widespread and run across hospital groups.^17^

Regardless of the sector, if interventions are accompanied with education and ongoing audits, ASPs would be successful and sustainable.^10^ One of the key differences between the public and private sector is the availability of electronic health records. This allows for easy retrieval of information in order to audit the impact of interventions on antibiotic use and consumption. Both are important measures to the success of a stewardship programme. Access to electronic health records is increasingly been realised as an important requirement for the implementation of more advanced AMS initiatives, especially in LMICs.^14,16,18^ Access to electronic health records having an inbuilt clinical decision support system (CDSS) is listed as one of the core global requirements for hospital AMS.^19^ Evidence suggests that an adequate human resource complement as well as ongoing funding are imperative to the sustainability and success of a programme. Few countries have established staffing norms for ASPs.^14^ Effective facilitation of AMS requires salary support and dedicated time.^14,19,20,21,22^ Pulcini et al. highlighted the need for a global list of core activities and essential resources including diagnostics and pharmacy support that are required to establish an ASP.^14^ These include ‘minimum international staffing standards for stewardship teams’ (p. 3).^14^

In the wake of the COVID-19 pandemic, stewardship advocacy has become even more critical. It is vital to put in place the measures to curtail unnecessary use of antibiotics and restrict their use to those patients with the most severe COVID-19 symptoms.^7^ It is imperative that dedicated allocation of funding for stewardship programmes be made. This should be part of the patient safety and quality improvement budget.^14^ The only way forward for AMS progression is the deliberate investment into the programme made with regards to staff, time, IT, and resources.
